# Supplementary figures and images for: Mutations that confer resistance to broadly-neutralizing antibodies define HIV-1 variants of transmitting mothers from that of non-transmitting mothers
Source: PLoS Pathog. 2021 Apr 2;17(4):e1009478. doi: 10.1371/journal.ppat.1009478 (PMC8055002; doi:10.1371/journal.ppat.1009478)

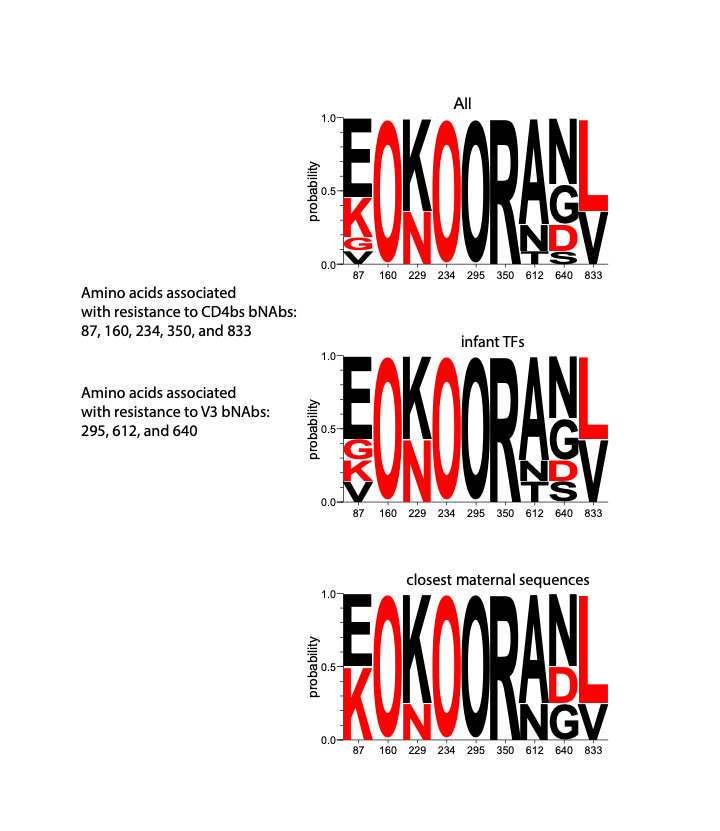

Supplement: S1 Fig — Logo plots of infant TF sequences, and their paired closest maternal sequences at 9 sites that were found to be associated to changes in bNAb sensitivity in the remaining Envs from transmitting and non-transmitting mothers (Table 2). Each stack shows the different amino acids at a single Env position, with letters proportional to the frequencies at which they were found in the sequence sample. Red indicates residues associated with resistance, and O indicates an N-linked glycosylation site. Top: Logo plot of all infant TF and paired closest maternal sequences, combined. Middle: infant TF sequences only. Bottom: maternal sequences closest to infant TFs. All logo plots were obtained using the AnalyzeAlign tool available on the LANL database (https://www.hiv.lanl.gov/content/sequence/ANALYZEALIGN/analyze_align.html). (TIFF) [file ppat.1009478.s004.tiff]
